# News and Coming Events

**Published:** 1908-11-21

**Authors:** 


### NEWS AND COMING EVENTS.

THE funeral of Sir Henry Pitman took place at Enfield on November 12. Sir Richard Douglas Powell, President of the Royal College of Physicians, attended, and the Royal College of Surgeons, St. George's Hospital, and other medical institutions were also represented.

THE Bradshaw Lecture before the Royal College of Surgeons of England will be delivered by Sir W. Watson Cheyne, Bart., on December 4. Mr. G. H. Makins, C.B., has resigned his seat on the Court of Examiners, and the vacancy will be filled up next month. Mr. A. W. Mayo Robson has been elected the representative of the College on the Court of Governors of the University of Sheffield.

THE King has been pleased, upon the recommendation of the Secretary for Scotland, to appoint Mr. George Dean, M.B., C.M., Chief Bacteriologist at the Lister Institute of Preventive Medicine, to the chair of Pathology in the University of Aberdeen, vacant by the resignation of Professor D. J. Hamilton.

EARL AMHERST presided at the half-yearly general meeting of the British Home and Hospital for Incurables held at the institution, Crown Lane, Streatham, on November 12. The Chairman, in appealing for increased subscriptions and new subscribers, stated that during the half-year ending October the subscriptions and donations had amounted to £2,667, as compared with £7,487 in the corresponding period last year, showing a deficit of £4,820.

IN memory of the late Mr. Alfred Boyd, of Sandon, Essex, an ex-member of the Canadian Parliament, his widow has given £1,000 to St. Bartholomew's Hospital to provide the scientific instruments for the new pathological laboratories. One of the laboratories is to be named the Alfred Boyd Memorial Laboratory, and the hospital authorities have decided to invite the Lord Mayor to open the new pathological wing at an early date.

THE Secretary of State for the Colonies has appointed a Departmental Committee to inquire into the duties, organisation, emoluments, and selection of officers of the West African Medical Staff. The Committee will be composed of: Mr. H. J. Read, C.M.G., of the Colonial Office, Chairman; Dr. T. Thomson, C.M.G., of the Local Government Board; Dr. W. H. Langley, C.M.G., Principal Medical Officer of the Gold Coast; Dr. J. K. Fowler, Dean of the Faculty of Medicine in the University of London; and Mr. A. Fiddian and Mr. H. C. W. Verney, of the Colonial Office. Mr. J. R. W. Robinson, of the Colonial Office, will act as secretary to the Committee.

THE Bishop of Kensington, on November 15, dedicated an altar at the West End Hospital for Nervous Diseases, Bulstrode Street, W. The altar, which is placed in the largest of the wards, is, with the ornaments and communion plate, a gift to the hospital by Mr. Ian Malcolm (chairman of the committee) and Mrs. Malcolm. After the dedication the Bishop delivered an address, in the course of which he said that the hospitals of London spoke sermons which no human eloquence could rival. The hospital premises in Bulstrode Street contain twenty-five beds for adults, and those in Welbeck Street fifty beds for children. The committee hope to be able to construct next year in Bulstrode Street a new hospital, which will contain one hundred beds for infant and adult patients, thus securing greater efficiency in treatment and more economy in management.

MR. EDWARD WHISHAW HENLEY, M.R.C.S., L.R.C.P., medical superintendent of Gloucestershire County Asylum, died at Gloucester on Saturday afternoon, after an operation for appendicitis.

THE Salters' Company have given an annual subscription of £10 10s. to the funds of the After-Care Association for Poor Persons discharged recovered from Asylums for the Insane, Church House, Westminster.

MR. SOMERVILLE HASTINGS, F.R.C.S., Mr. J. C. Mottram, M.B., and Mr. Bryden Glendinning, M.B., B.S., have been appointed to the "Salters' Company," the "Richard Hollins," and the "Walter Emden" scholarships respectively, in the Cancer Research Laboratories at the Middlesex Hospital.

AT the meeting of the quarterly general board of Westminster Hospital, to be held on November 24, Mr. James Hora will be proposed for election as joint treasurer of the hospital, in succession to the late Mr. C. A. R. Hoare. Mr. Hora is a vice-president of the hospital and a generous contributor to its funds. He gave largely to the endowment of the Marie Celeste ward for children and of the Marie Celeste Samaritan Society for the assistance of necessitous patients on their discharge.

THE Glasgow University Club for Manchester and District will hold its third annual dinner at the Midland Hotel, Manchester, on Wednesday, December 2, when the principal guests of the evening will be Principal Sir Donald MacAlister, K.C.B., Miss Galloway (Queen Margaret College), and Professors Gemmell, Ferguson, and Freeland Fergus. Further particulars can be obtained from the Secretary, Dr. D. Richmond, 176 Drake Street, Rochdale, who will be glad to receive the name and address of any graduate in the district who may not have received a notice by post.

IN the House of Commons Mr. P. O'Brien recently asked the First Commissioner of Works whether he was yet in a position to state what arrangements he had been able to make with a view to secure that the necessary medical and surgical appliances were available, and a medical man to apply them, in case of sudden illness occurring within the premises of the House. Mr. L. Harcourt replied that a large medicine chest, replete with every drug and instrument which the ingenuity or necessities of man could require, had been generously presented to the House by a well-known firm of manufacturing chemists, who did not desire their name to be mentioned in the matter.

THE Asylums Committee of the London County Council have received from Dr. J. J. Nason, of Stratford-on-Avon, through the librarian of Birmingham University, an engraving from a portrait of Dr. John Conolly, who was medical superintendent of Hanwell Asylum from 1839 to 1844, and visiting physician from 1844 until 1852. The engraving is after Sir Watson Gordon's portrait, which was presented to Dr. Conolly as a public testimonial together with a piece of plate on the resignation of his appointment. The committee state that they are pleased to possess a momento of the distinguished man who established at Hanwell Asylum, on a scale hitherto unprecedented, the humane system of treatment of the insane.

Dr. J. Sholto Cameron Douglas, eldest son of Dr. Claude Douglas, of Leicester, who in July last rescued a lady from drowning at Trevone Bay, has been awarded the Royal Humane Society's medal.

Under the will of the late Miss Annie Scott, of Chester Square, W., the Brompton Hospital for Consumption, the Brompton Cancer Hospital, and the Grosvenor Hospital for Women and Children each receive a sum of £5,000.

The Worshipful Company of Barbers will celebrate the six hundredth anniversary of the admission of the first recorded Master of the Company, Richard le Barbour, who became Master in 1308, by a dinner, which will be held at Grocers' Hall on Tuesday, December 15, and a large number of distinguished guests have been invited, including the most prominent members of the medical and surgical professions.

The death of Victorien Sardou, the great damatist, recalls the fact that his father, Professor Antoine Léandre Sardou, a Provençal, intended him for the medical profession. Sardou actually commenced to study with a view to qualifying as a doctor, but the family funds became exhausted, and young Sardou, whose tastes had never been strongly in the direction of medicine, was for some five or six years in a condition of penury.

At the Liverpool Town Hall on November 6 Dr. Richard Caton (the Lord Mayor of Liverpool) was presented with his bust in marble, which had been subscribed for by all his fellow members of the City Council. The ceremony was attended by members of the Council and a few personal friends. Sir William Forwood made the presentation, and spoke in the warmest terms of the good work of the Lord Mayor on behalf of the city and the port.

Much damage was done by fire at the Royal Albert Hospital, Devonport, on November 14. The wooden extension of the main building over the front entrance, where some painters had been working, caught fire, and fanned by a high wind the flames speedily spread over the front of the hospital. The men's and women's wards, which immediately adjoin the extension and occupy separate floors, were filled with smoke, and great excitement prevailed among the patients. The coolness and promptitude of the hospital staff, however, enabled the whole of the patients to be removed to another wing of the block, and meanwhile the local fire brigade was summoned. The fire was eventually extinguished, and the main buildings were saved.

The Local Government Board issued the following notice on November 13 : "Altogether three cases of plague have occurred in Liverpool between October 20 and November 6. These cases were confined to a barge in the docks, the last patient having been taken to hospital and isolated on the 6th inst. Every precaution, including isolation of the patients, watching of all known contacts, and disinfection, has been taken. A large number of rats have been killed in the docks and examined in the municipal laboratory. No instance of plague in any of these rats has been found. No further cases of plague have developed since the 6th inst., and under Article 9 of the Paris Convention of 1903, any port which has become infected by plague ceases to be infected five days after the isolation or the death or the recovery of the last case of plague and after all measures of disinfection have been carried out and measures against rats have been taken."

Mr. George Waugh, B.A. Cantab., M.D., B.S. Lond., F.R.C.S. Eng., has been appointed surgeon to out-patients at the Hampstead General Hospital.

The Lady Mayoress will open the annual Christmas sale of articles made by the members of the Children's Salon in the Ball Room of the Hyde Park Hotel on Tuesday, December 15. The proceeds will be devoted to the endowment of the Salon's ninth cot—on this occasion in the Evelina Hospital for Sick Children, Southwark.

At Lewes recently, before Mr. Justice Grantham, the trial concluded of Charles Montague, sixty-two, described as a "rheumatic specialist," who was indicted for obtaining money by false pretences with intent to defraud. The prisoner sold what purported to be a cure for rheumatoid arthritis, and this was alleged to be absolutely useless. The prisoner had been under the notice of the Metropolitan Police since 1883 as a medicine vendor, and they had received complaints concerning the sale of medicines by him. From 1900 to 1906 the prisoner carried on the business of a so-called rheumatic specialist at Birmingham. Investigations showed that in one instance testimonials were given by a lady typist in the prisoner's employ. The Judge said the prisoner acted wisely in pleading guilty to attempting to obtain money by false pretences. His Lordship said the remedies were quack remedies. No doubt the prisoner had been making large profits and it was fortunate he had been exposed. He passed sentence of six months' imprisonment.

At the Guildhall, before Alderman Sir Vezey Strong, "Dr." Henry Leins, of 149 Houndsditch, was summoned under the Sale of Food and Drugs Act for selling a compounded drug which was not composed of the ingredients demanded by the purchaser. The subject was certain opium and lead pills, four of which were certified by the Public Analyst to be deficient in lead acetate. Formal evidence was given to the effect that a prescription was left at defendant's shop in accordance with which a dozen pills were made up. Four were submitted to the Public Analyst, four left with the defendant, and the remainder were produced in court. Mr. Kirby, who defended, objected to the insufficiency of the analysis and certificate. Only four pills out of twelve were analysed, and the deficiency in those might easily have been good in the others.

Mr. Vickery (prosecuting for the Corporation) said that those four were being dealt with and did not come up to the standard required by the British Pharmacopœia.

The following discussion then took place, as reported in *The Times* :

Sir Vezey : But supposing the other eight pills contained a *plus* quantity of the drug in question at the expense of the four actually analysed?

Mr. Vickery : I should say it was a most serious thing.

Sir Vezey : Do you suggest that it is possible, in mixing up a mass of material, to do so in such a manner that a twelfth shall contain precisely all the percentages of the ingredients which should characterise the whole mass?

Mr. Vickery : Certainly, and I think it would be very unfortunate if the public faith in the chemist was shaken in that direction.

Sir Vezey : I do not think we have got sufficient particulars to warrant a conviction. I shall dismiss the summons on the ground that the certificate does not sufficiently disclose the particulars. In fact, it does not comply with the statute.

Mr. Vickery said he thought the effect of this decision would be to disturb the public faith in chemists supplying pills of uniform strength and quality.

